# Incidence of adverse events in a Brazilian coronary ICU

**DOI:** 10.1186/cc13195

**Published:** 2014-03-17

**Authors:** VF Paula, CE Bosso, OA Souza, GE Valerio, SV Ferreira

**Affiliations:** 1 Instituto do Coraçao de Presidente Prudente, Brazil; 2 UNOESTE - Universidade do Oeste Paulista, Presidente Prudente, Brazil

## Introduction

Security policies for the patient are an interest to any health professional. The rates of adverse events in hospitals reach values ranging between 3.7 and 16.6%, being the highest range (40 to 70%) considered preventable or avoidable [1]. The objective of this study is to analyze the incidence of adverse events and their severity in a Brazilian coronary ICU (CICU).

## Methods

Research conducted from database analysis regarding hospitalizations from 1 May 2012 until 31 October 2013 in a coronary care unit of the city of Presidente Prudente, Brazil. Statistical analysis was performed using EPI INFO, version 3.5.2 software, which was considered significant at *P *< 0.05 two-tailed and CIs at 95% (CI) were used for the odds ratios (OR) estimated in the sample. Demographics (gender and average age) and aspects related to adverse events were analyzed, the latter being under the impact and probability of death.

## Results

A total of 1,067 admissions were analyzed in the period, with 57.38% male and 42.63% female. The average age was 67.7 ± 13.2 years. The average CICU length of stay was 3.74 ± 5.51 days. A total of 211 adverse events occurring in 140 different admissions were recorded. The most frequent were drug administration errors (24.3%), pressure ulcer (24.3%), phlebitis (22.1%), loss of enteric tube (13.6%) and central venous cannulation accident (7.1%). The statistical analysis shows that hospitalization time longer than 2 days are related to the occurrence of the events (OR: 8.08 CI: 4.95 to 13.2) and that the presence of the first occurrence is significant to increase the probability of death in the unit (OR = 5.33, CI: 3.49 to 8.12). Pressure ulcer (OR = 16.73, CI: 8.04 to 34.81), enteral tube loss (OR 3.64, CI: 1.36 to 9.75) and drug administration errors (OR = 2.87, CI: 1.31 to 6.31) were also related to higher mortality.

## Conclusion

The research draws attention to a significant incidence of adverse events and shows that their occurrence implies an increase of death in the unit. Therefore, security measures should be employed for the reduction, and thus enhance the quality of service provided by health professionals to patients admitted to a coronary care unit.
